# Correlation between plasmacytoid dendritic cell activation and suppression of subjective physical symptoms following *Lactobacillus paragasseri* SBT2055 ingestion: a randomized, double-blind, placebo-controlled, parallel-group comparative study

**DOI:** 10.3389/fnut.2025.1722081

**Published:** 2026-01-12

**Authors:** Eiji Kobatake, Shukuko Ebihara, Masaya Miyoshi, Toshiharu Namba, Toshinobu Arai

**Affiliations:** 1 Milk Science Research Institute, Megmilk Snow Brand Co., Ltd., Kawagoe, Japan; 2 Chiyoda Paramedical Care Clinic, Tokyo, Japan; 3 Research and Development Planning Department, Megmilk Snow Brand Co., Ltd., Tokyo, Japan; 4 CPCC Company Limited, Tokyo, Japan

**Keywords:** clinical study, common cold, *Lactobacillus paragasseri* SBT2055, plasmacytoid dendritic cells, subjective symptoms

## Abstract

**Objective:**

This study examined the relationship between physical condition and plasmacytoid dendritic cell (pDC) activation in healthy adults who ingested *Lactobacillus paragasseri* SBT2055 (LG2055).

**Methods:**

This randomized, double-blind, placebo-controlled, parallel-group comparative study involved 200 participants who were randomly divided into the LG2055 and placebo groups. The participants ingested one bottle of drinkable yogurt with or without LG2055 daily for 12 weeks. A daily physical health questionnaire on local and systemic symptoms of the common cold was used as the primary outcome. The secondary outcomes were pDC activity, pDC frequency, and IFN-α production by peripheral blood mononuclear cells.

**Results:**

Of the 200 participants, 196 completed the intake, and after excluding 8 participants, 188 (95 in the LG2055 group and 93 in the placebo group) were analyzed. The LG2055 group showed a significantly lower ratio of “with symptoms” during the intake period for nine symptoms (nasal congestion, sneezing, hoarseness, cough, headache, general malaise, chills, feverishness, and feeling unwell) (Chi-squared test, *p* < 0.05). Based on reports that aging correlates with numerical and functional decline in pDCs, stratified analysis was conducted and revealed that changes in the expression levels of cell surface markers (CD86, HLA-DR, and CD40) in pDCs were significantly higher in the LG2055 group among young participants (<40 years), whereas LG2055 intake suppressed changes in physical condition. In middle-aged participants (≥40 years), LG2055 neither activated pDCs nor strongly influenced their physical condition.

**Conclusion:**

The study results indicate that pDC activation by LG2055 contributes to the maintenance of physical condition and suggests that LG2055 improves subjective symptoms by activating pDCs.

**Clinical trial registration:**

https://www.umin.ac.jp/ctr/index.htm, identifier UMIN000055950.

## 1 Introduction

The human immune system is a defense mechanism to protect themselves from non-self entities, such as foreign substances and pathogens. The immune system comprises innate immunity, which is a non-specific response, and adaptive immunity, which is a specific response to pathogens. Innate and adaptive immunity play distinct roles in host defense. These systems must function properly to maintain host homeostasis; thus, failure or imbalance in these systems can induce host illness.

Some beneficial microbes, such as probiotics and postbiotics, have been reported to modulate immune systems. Probiotics have been defined as “live microorganisms that, when administered in adequate amounts, confer health benefits on the host” in a report by a joint FAO/WHO working group (1). In contrast, the International Scientific Association for Probiotics and Prebiotics has defined postbiotics as a “preparation of inanimate microorganisms and/or their components that confers a health benefit on the host” (2). Some studies have suggested that probiotics and postbiotics exert their effects via the intestinal immune system. The intestinal tract ingests and absorbs orally ingested substances and is constantly exposed to foreign substances; therefore, countermeasures against harmful pathogens and substances are essential. The intestinal immune system comprises lymphoid tissues and various immune cells that work together to activate innate and adaptive immunity, resulting in host protection (3). Probiotics and postbiotics are considered safe food ingredients. Simultaneously, they are foreign substances to the host and can safely stimulate the intestinal immune system of the host. Consequently, they induce an immune response via the intestinal immune system and enhance host resistance to pathogens.

*Lactobacillus paragasseri* SBT2055 (LG2055) is a strain of lactic acid bacteria (LAB). LG2055, formerly classified as *Lactobacillus gasseri*, was reclassified as *L. paragasseri* in 2018 (4, 5). It has been used as a probiotic LAB in the commercial manufacture of dairy products for over two decades. Numerous studies have reported the immunostimulatory effects of LG2055; thus, LG2055 is expected to be applied as an immune-stimulating food ingredient. For example, enhanced immunoglobulin A (IgA) production and protection against viral infections have been demonstrated *in vivo* following LG2055 administration (6–8). In addition, a clinical study revealed that the intake of LG2055 resulted in increased production of salivary secretory IgA, serum IgA, and IgG; upregulation of natural killer cell activity; increased antiviral gene expression; and elevated hemagglutination inhibition titers against the influenza virus after vaccination (9). Our *in vitro* study suggested that intestinal LG2055 is translocated by M cells in Peyer’s patches and phagocytosed by plasmacytoid dendritic cells (pDCs), resulting in pDC activation (10). pDCs are important immune cells that play key roles in antiviral responses. pDCs are activated by viral stimulation, and activated pDCs produce type I interferons while activating immune cells such as natural killer cells, B cells, and T cells. Consequently, pDC activation induces an overall immune response, protecting the host from viral infections (11). Our previous clinical studies revealed that the intake of LG2055 significantly suppressed the cumulative days of subjective common cold-like symptoms during the intake period compared with that with the placebo (5, 12). In addition, LG2055 induced pDC activation, especially in healthy individuals who showed salivary secretory IgA secretion rates under the average in the participants, and suppressed common cold-like symptoms (12). These results suggest that pDC activation induced by LG2055 intake contributes to the suppression of subjective common cold symptoms in healthy adults; however, investigations into the relationship between the maintenance of physical condition and pDC activation in healthy adults who ingested LG2055 remain insufficient and unclear. Therefore, in this study, we conducted an additional clinical trial to investigate the relationship.

## 2 Materials and methods

### 2.1 Study design

The study design was based on those of previous clinical studies (5, 12). This clinical trial was conducted from October 2024 to April 2025 as a randomized, double-blind, placebo-controlled, parallel-group, and comparative study. The study protocol was approved by the Institutional Review Board of Chiyoda Paramedical Care Clinic on October 18, 2024 (approval number: 24101801). The approved protocol was registered in the University Hospital Medical Information Network Clinical Trials Registry before the start of the trial (UMIN ID: UMIN000055950). To protect the participants’ human rights, this study was conducted according to the principles of the Helsinki Declaration of 1975 (revised in 2013), the Ethical Guidelines for Medical and Health Research Involving Human Subjects (partially revised in 2023), and the approved protocol.

This study comprised a screening test, a 1 week pre-observation period, and a 12 week intake period.

### 2.2 Participants

Healthy Japanese males and females aged 20–64 years who had the capacity to understand the details of this study and provide consent of their own free will were recruited. They identified themselves as individuals who could easily develop a cold. Individuals who met any of the exclusion criteria were excluded (Supplementary Table 1). The participants were instructed to follow the instructions during the study (Supplementary Table 2). All participants received a thorough explanation of the study purpose and details and provided written informed consent before their participation.

### 2.3 Test samples

Test samples, active drinkable yogurt (DY) containing LG2055, and placebo DY lacking LG2055 were prepared as described in our previous studies (5, 9). Briefly, approximately 10% dairy products and small amounts of flavoring agents, stabilizers, and artificial sweeteners were mixed and pasteurized to prepare the DY mixture. The obtained DY mixture was inoculated with LG2055 and *Streptococcus thermophilus* (commercially available yogurt starter cultures) and fermented to obtain active DY. The fermentation process was monitored by measuring titratable acidity and pH. The placebo DY was prepared in the same manner but without inoculation with LG2055. Active DY contained at least 1 × 10^9^ colony-forming units/100 g of LG2055 per bottle, whereas LG2055 was not detected in the placebo DY. The active and placebo DY had the same nutritional composition (energy: 36 kcal, protein: 3.3 g, fat: 0 g, carbohydrate: 5.5 g, sodium: 43 mg/100 g) and were indistinguishable in terms of taste, flavor, and appearance. The prepared test samples were stored in cold storage and delivered to the participants weekly. The participants ingested one bottle (100 g) of active or placebo DY once daily during the 12 week intake period.

### 2.4 Outcomes

The primary outcome resulted from a daily physical health questionnaire survey on local and systemic symptoms associated with the common cold. The secondary outcomes were pDC activity, pDC frequency, and interferon-α (IFN-α) production from peripheral blood mononuclear cells (PBMCs).

The daily physical health questionnaire was designed according to our previous clinical trials (5, 12) and other studies (13–15) (Supplementary Figure 1). Participants evaluated and recorded the severity of 12 subjective symptoms on a five-point scale (no symptoms, very mild, mild, moderate, and severe) using a questionnaire daily during the screening, pre-observation, and intake periods. The evaluated symptoms comprised seven local symptoms (runny nose, nasal congestion, sneezing, sore throat, hoarseness, cough, and headache) and five systemic symptoms (general malaise, chills, feverishness, fatigue, and feeling unwell). Based on the questionnaire results, the cumulative number of days with incidences was calculated and evaluated for each symptom. A questionnaire survey was administered in the Japanese language.

PBMCs were obtained from blood samples and frozen until further analysis. To measure pDC activity, frozen PBMCs were thawed, treated with HumanTruStain FcX™ (Fc Receptor Blocking Solution) (BioLegend, San Diego, CA, United States), and stained with fluorescent dye-conjugated antibodies. The following antibodies were used: FITC anti-human CD123, APC anti-human BDCA-4, PE anti-human CD86, PerCP/Cy5.5 anti-human HLA-DR, PE anti-human CD80, and PerCP/Cy5.5 anti-human CD40 (BioLegend). The stained cells were washed with 1% fetal bovine serum-DPBS, and fluorescence was measured using a BD FACSVia™ flow cytometer (BD, Franklin Lakes, NJ, United States). Data were analyzed using the BD FACSVia™ Research Software (BD). In this study, CD123^+^ BDCA-4^+^ cells were defined as pDCs (12). The expression levels of the cell surface markers (CD86, HLA-DR, CD80, and CD40) on pDCs were used to represent pDC activity, and the mean fluorescence intensity (MFI) was measured. Isotype controls were used to confirm the specificity. Concurrently, the percentage of pDCs in monocytes, gated using forward/side scatter, was determined; that number represented the pDC frequency. The pDC activity and frequency were measured during screening and at 0, 4, 8, and 12 weeks after starting the intake.

To evaluate IFN-*α* production, frozen PBMCs were thawed and resuspended in RPMI 1640 medium (Sigma-Aldrich, St. Louis, MO, United States) supplemented with 1 mM sodium pyruvate (Thermo Fisher Scientific, Waltham, MA, USA), 2.5 mM HEPES (Thermo Fisher Scientific), 100 U/mL penicillin-streptomycin (Nacalai Tesque, Kyoto, Japan), 50 μM 2-mercaptoethanol (Sigma-Aldrich), and 10% fetal bovine serum. PBMCs were seeded at 5 × 10^5^ cells/500 μL/well in a 48-well plate and treated with 1.0 μg/mL Influenza A H1N1 Antigen (Hytest Ltd., Turku, Finland) for 24 h at 37 °C and 5% CO_2_. Culture supernatants were collected and stored at −80 °C until they were measured. The concentration of IFN-*α* in the supernatant was measured using the VeriKine-HS Interferon α All Subtype TCM ELISA Kit (PBL Assay Science, Piscataway, NJ, United States) and calculated from calibration curves according to the manufacturer’s instructions.

### 2.5 Safety assessment

During the study, adverse events and changes in clinical examination scores were evaluated for safety. The participants recorded their test sample consumption, diet, alcohol consumption, exercise, defecation, sleep, menstruation, body temperature, intake of restricted foods, subjective physical condition, visits to medical institutions, medical treatments, medication use, and vaccinations during the pre-observation and intake periods.

The participants received a doctor’s consultation, and subjective and objective symptoms were determined during the screening test and at 0, 4, 8, and 12 weeks after starting intake. At the same time, body weight, body mass index (BMI), blood pressure, pulse, hematological parameters (white and red blood cell counts, hemoglobin, hematocrit, and platelets), biochemical parameters (total protein, albumin, total bilirubin, alkaline phosphatase, lactate dehydrogenase, aspartate aminotransferase, alanine aminotransferase, gamma-glutamyl transpeptidase, creatine kinase, total cholesterol, triglycerides, high-density lipoprotein cholesterol, low-density lipoprotein cholesterol, urea nitrogen, creatinine, uric acid, sodium, potassium, chloride, calcium, and glucose), and urinalysis parameters (protein, glucose, urobilinogen, bilirubin, and occult blood) were measured.

Adverse events were defined as any undesirable or unintended injury or illness, including abnormal changes in clinical examination scores. Adverse events that occurred during the intake period were also recorded. When any adverse event occurred, the details of the adverse event were surveyed, and a medical doctor judged the severity and causal relationship with the intake of the test samples.

### 2.6 Sample size

The sample size was determined using data from a previous study (5). Based on the cumulative number of days of total subjective symptoms in the study, more than 84 participants in each group were required to detect a difference between the LG2055 and placebo groups at a 5% significance level and a statistical power of 80%. Considering a dropout rate of 10%, 100 participants were included in each group of the study.

### 2.7 Randomization

The participants were equally divided into two groups according to block randomization based on sex and age. An independent controller assigned each participant to the LG2055 or placebo group. The assignment list was sealed by the controller until the designated unmasking time was reached.

### 2.8 Statistical analysis

For the primary outcome, the severity of each symptom in the daily physical health questionnaire results was converted into two grades: “without symptoms” and “with symptoms,” in accordance with our previous clinical trials (5, 12). Briefly, “without symptoms” included “no symptoms,” whereas “with symptoms” included “very mild, mild, moderate, and severe” on a five-point scale. The cumulative number of days of incidence was calculated for each of the symptoms. The cumulative days of symptoms were counted for each group and analyzed using the chi-squared test.

For the secondary outcomes and safety assessments, the change from baseline (0 weeks) to each subsequent time point (4, 8, and 12 weeks) was calculated for immune parameters. The averages and standard deviations (SD) were calculated for each marker based on the measured values and changes from baseline. For between-group comparisons, Welch’s *t*-test was used to analyze parametric parameters, whereas the Wilcoxon rank-sum test was used to analyze nonparametric parameters. Statistical analyses were performed using IBM SPSS Statistics (version 30.0; IBM Corp., Armonk, NY, United States). Statistical significance was set at *p* < 0.05.

In addition, a stratified analysis by age was conducted. The statistical analysis plan, including the stratified analysis, was pre-defined before the study was unblinded.

## 3 Results

### 3.1 Participants

**Figure 1:**
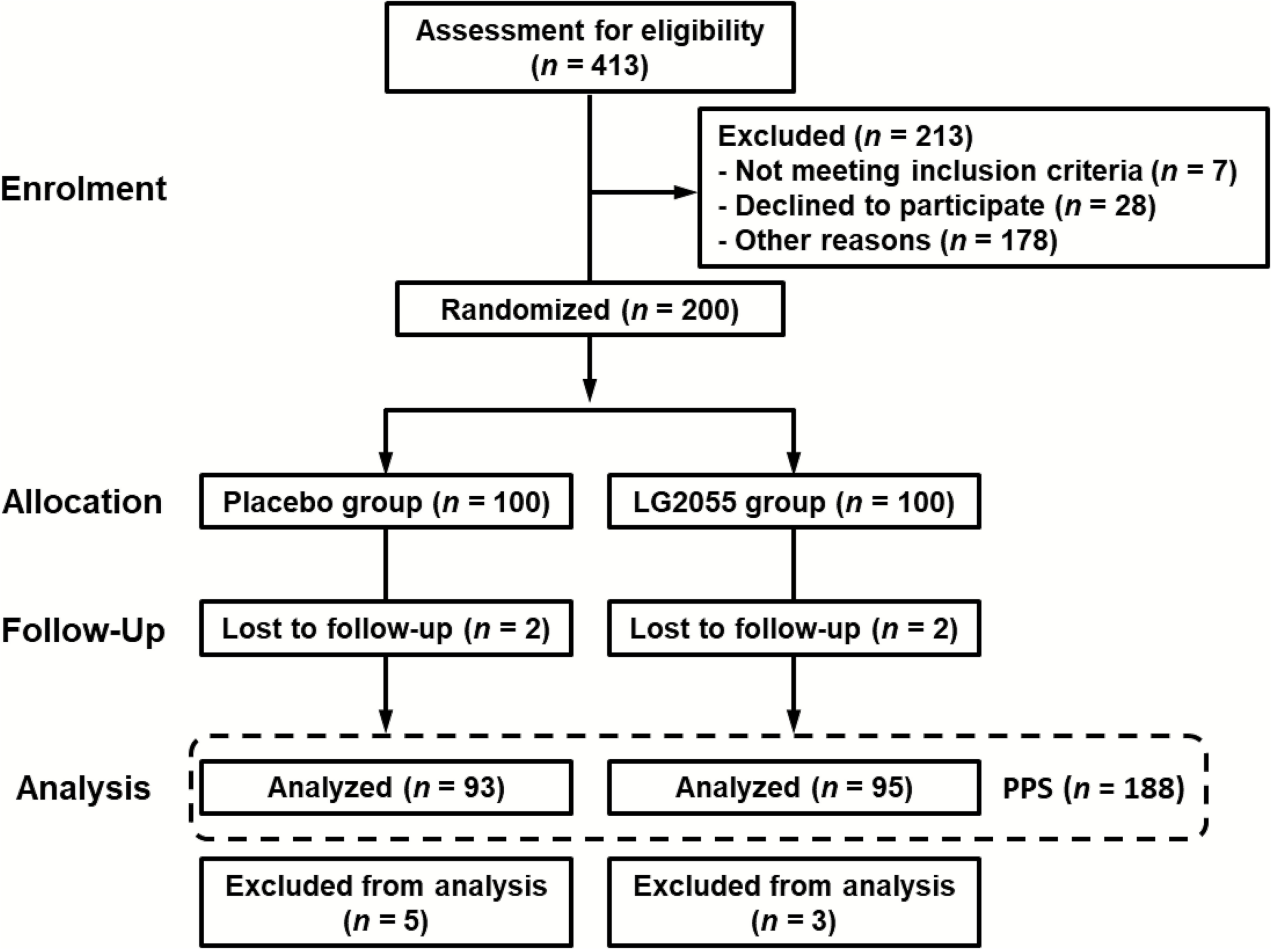
Flowchart of the participant selection process.

A flowchart of the participant selection process is shown in Figure 1. Participant recruitment for this study began in October 2024. A total of 200 participants were selected from 413 candidates based on their suitability and enrolled in this study. The participants were randomly divided into two groups (LG2055 and placebo), with 100 participants in each group. Each test sample (active or placebo) was administered to the participants. The participants’ background characteristics are shown in Table 1. The total protein level was significantly different between the two groups (7.27 ± 0.36 g/dL in the LG2055 group vs. 7.44 ± 0.38 g/dL in the placebo group, Welch’s *t*-test, *p* = 0.001); however, the difference between two groups was slight and the values were within the normal range. Thus, it was not considered to affect the results by a medical doctor. No significant differences in any other parameters were observed between the two groups, and the levels of hematological and biochemical markers were within the normal range for each group. Based on the daily physical health questionnaire, the cumulative number of days with the sum of each symptom was not significantly different between the two groups during screening tests.

**Table 1:** Background characteristics of the participants in each group.

		Placebo group	LG2055 group	*p*-value
		*n* = 100	*n* = 100	
Sex	Male/female	45/55	46/54	1.000
Age	Years	45.7 ± 12.7	45.6 ± 11.7	0.972
Height	cm	164.0 ± 8.3	164.3 ± 7.8	0.843
Weight	kg	58.4 ± 11.3	57.8 ± 10.6	0.706
BMI	kg/m^2^	21.5 ± 2.8	21.3 ± 2.6	0.484

Values are presented as the mean ± SD.

During the intake period, two participants declined to participate because of physical problems (appendicitis and body itching). In addition, two participants were diagnosed with suspected peritonitis or diverticulitis and discontinued the study at the investigator’s discretion; thus, 196 participants completed the scheduled intake of the test samples. Subsequently, eight participants were excluded from the efficacy analysis due to the intake of medications, such as antibiotics and anti-allergy medications, which could have influenced the outcome, and due to vaccinations during the study period. Consequently, 188 participants were included in the per-protocol set (PPS) for efficacy analysis (95 in the LG2055 group and 93 in the placebo group).

Among the 196 participants who completed the scheduled intake of test samples, the intake rate was >94.0%. The average intake rate among all participants was 98.4% in the LG2055 group and 98.5% in the placebo group. No significant difference was observed in the intake rates between the two groups (Welch’s *t*-test).

### 3.2 Primary outcome

A comparison of the cumulative number of days for each symptom is shown in Table 2. In comparison with the placebo group, the LG2055 group showed a significantly lower ratio of “with symptoms” during the intake period (1–12 weeks) for nine symptoms (nasal congestion, sneezing, hoarseness, cough, headache, general malaise, chills, feverishness, and feeling unwell). In the short-term evaluation, the LG2055 group showed a significantly lower ratio of “with symptoms” for seven symptoms (nasal congestion, hoarseness, cough, headache, general malaise, feverishness, and feeling unwell) during 1–4 weeks, as well as for nine symptoms (nasal congestion, sneezing, hoarseness, cough, headache, general malaise, chills, feverishness, and feeling unwell) during 1–8 weeks (Table 2). These results indicate that the incidence rates of common cold-like symptoms were lower in the LG2055 group, and this trend was observed within 4 weeks of starting the ingestion.

**Table 2:** Comparison of the cumulative days of each symptom during the intake period.

Symptoms	Group	1–4 W			1–8 W			1–12 W		
		Without	With	*p*-value	Without	With	*p*-value	Without	With	*p*-value
Runny nose	Placebo	2,187	417	0.026^*^	4,277	931	0.419	6,482	1,330	0.248
	LG2055	2,172	488		4,336	984		6,565	1,415	
Nasal congestion	Placebo	2,277	327	0.006^*^	4,517	691	0.004^*^	6,756	1,056	<0.001^*^
	LG2055	2,390	270		4,714	606		7,066	914	
Sneezing	Placebo	2,392	212	0.256	4,579	629	<0.001^*^	6,789	1,023	<0.001^*^
	LG2055	2,466	194		4,797	523		7,128	852	
Sore throat	Placebo	2,323	281	0.258	4,667	541	0.848	7,056	756	0.471
	LG2055	2,346	314		4,774	546		7,180	800	
Hoarseness	Placebo	2,431	173	<0.001^*^	4,857	351	<0.001^*^	7,303	509	<0.001^*^
	LG2055	2,576	84		5,120	200		7,669	311	
Cough	Placebo	2,383	221	<0.001^*^	4,788	420	<0.001^*^	7,174	638	<0.001^*^
	LG2055	2,511	149		5,038	282		7,566	414	
Headache	Placebo	2,423	181	<0.001^*^	4,847	361	<0.001^*^	7,283	529	<0.001^*^
	LG2055	2,534	126		5,100	220		7,674	306	
General malaise	Placebo	2,421	183	0.036^*^	4,826	382	0.004^*^	7,266	546	<0.001^*^
	LG2055	2,511	149		5,004	316		7,544	436	
Chills	Placebo	2,504	100	0.066	4,997	211	<0.001^*^	7,546	266	<0.001^*^
	LG2055	2,583	77		5,178	142		7,795	185	
Feverishness	Placebo	2,544	60	<0.001^*^	5,075	133	<0.001^*^	7,628	184	<0.001^*^
	LG2055	2,631	29		5,251	69		7,875	105	
Fatigue	Placebo	2,328	276	0.253	4,695	513	0.447	7,065	747	0.428
	LG2055	2,404	256		4,820	500		7,247	733	
Feeling unwell	Placebo	2,511	93	0.019^*^	5,016	192	<0.001^*^	7,555	257	0.036^*^
	LG2055	2,595	65		5,186	134		7,763	217	

^*^Significant difference between the two groups (*p* < 0.05).

### 3.3 Secondary outcomes

**Figure 2:**
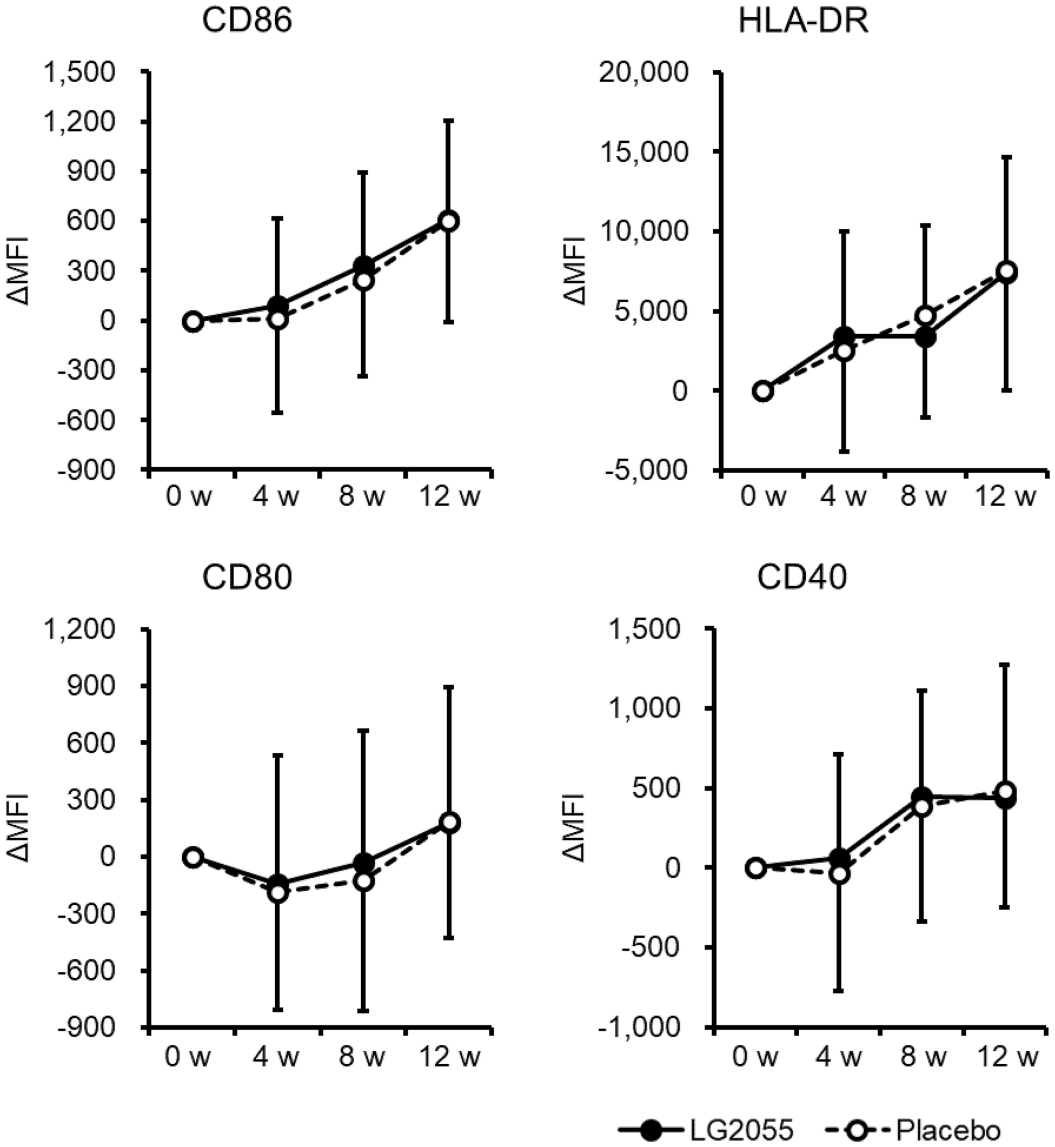
Comparison of changes in pDC activity from the baseline. PBMCs obtained from blood samples were stained and analyzed by flow cytometry. The expression levels of cell surface markers (CD86, HLA-DR, CD80, and CD40) on pDCs were used to represent pDC activity. Data are shown as the mean ± SD (LG2055 group, *n* = 95; placebo group, *n* = 93).

In the LG2055 group, parameters such as the changes from baseline in CD86, CD40, and IFN-α production remained slightly high during the intake period; however, there were no significant differences between the two groups in the measured values and the changes from baseline in CD86, HLA-DR, CD80, CD40, pDC frequency, and IFN-α production (Figure 2; Tables 3, 4; Supplementary Table 3).

**Table 3:** Comparison of pDC frequencies.

Week	Group	Mean ± SD (%)	*p*-value
0	Placebo	0.94 ± 0.69	0.560
	LG2055	1.00 ± 0.69	
4	Placebo	0.94 ± 0.55	0.920
	LG2055	0.95 ± 0.65	
8	Placebo	0.73 ± 0.46	0.209
	LG2055	0.83 ± 0.63	
12	Placebo	0.68 ± 0.45	0.363
	LG2055	0.74 ± 0.45	

**Table 4:** Comparison of IFN-α production.

Week	Group	Measured value (pg/mL)		Change from baseline (pg/mL)	
		Mean ± SD	*p*-value	Mean ± SD	*p*-value
0	Placebo	1,275.5 ± 1,025.8	0.585	—	—
	LG2055	1,205.1 ± 701.4		—	
4	Placebo	878.0 ± 488.8	0.796	−401.1 ± 948.6	0.467
	LG2055	900.0 ± 665.1		−305.1 ± 847.5	
8	Placebo	669.5 ± 369.7	0.528	−606.0 ± 989.6	0.749
	LG2055	637.5 ± 321.3		−567.6 ± 598.9	
12	Placebo	618.7 ± 271.7	0.811	−656.8 ± 1001.6	0.513
	LG2055	628.5 ± 293.8		−576.6 ± 627.9	

### 3.4 Stratified analysis

A stratified analysis was conducted to evaluate the influence of age on the effects of LG2055. Jing et al. (16) reported that aging correlates with numerical and functional decline in pDCs. They demonstrated that the frequency of pDCs in PBMCs and IFN-α production from pDCs were lower in healthy elderly subjects (age range: 64–92) than in healthy young subjects (age range: 21–40). According to this report, 188 participants with PPS were stratified into two layers: participants under 40 years (young participants: 28 participants in the LG2055 group and 32 participants in the placebo group, respectively) and participants aged 40 years and older (middle-aged participants: 67 participants in the LG2055 group and 61 participants in the placebo group, respectively). In each layer, there were no significant differences in the participants’ background characteristics between the LG2055 and placebo groups (Table 5).

**Table 5:** Background characteristics of the participants in each group (stratified analysis).

		Young participants (under 40 years)			Middle-aged participants (40 years and older)		
		Placebo group	LG2055 group	*p*-value	Placebo group	LG2055 group	*p*-value
		*n* = 32	*n* = 28		*n* = 61	*n* = 67	
Sex	Male/female	14/18	12/16	1.000	30/31	32/35	1.000
Age	Years	31.3 ± 4.8	29.5 ± 3.8	0.107	53.9 ± 7.1	52.0 ± 6.5	0.129
Height	cm	164.3 ± 8.0	165.8 ± 7.1	0.458	164.1 ± 8.6	163.6 ± 8.2	0.743
Weight	kg	56.9 ± 9.5	57.9 ± 10.2	0.685	59.8 ± 12.3	58.0 ± 11.0	0.368
BMI	kg/m^2^	21.0 ± 2.4	20.9 ± 2.4	0.969	22.0 ± 3.1	21.5 ± 2.7	0.294

Values are presented as the mean ± SD.

**Figure 3:**
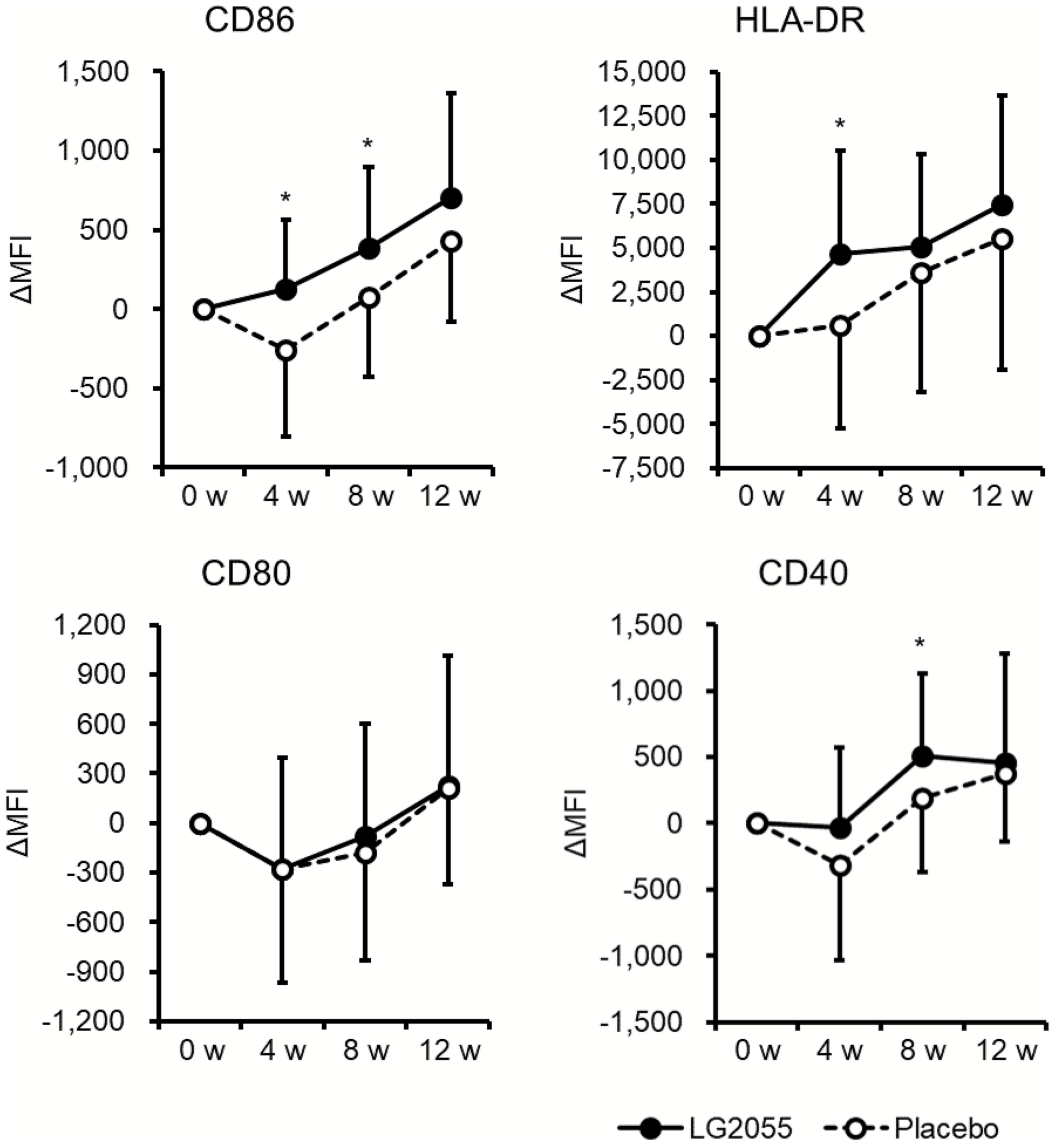
Comparison of changes in pDC activity from baseline (young participants). PBMCs obtained from blood samples were stained and analyzed by flow cytometry. The expression levels of cell surface markers (CD86, HLA-DR, CD80, and CD40) on pDCs were used to represent pDC activity. Data are shown as the mean ± SD. ^*^Significant difference between the two groups (*p* < 0.05) (LG2055 group, *n* = 28; placebo group, *n* = 32).

Among the young participants, the LG2055 group showed a significantly lower ratio of “with symptoms” during the intake period (1–12 weeks) for eight symptoms (sore throat, hoarseness, cough, headache, general malaise, chills, feverishness, and feeling unwell) compared with the placebo group, as well as for eight symptoms (runny nose, sore throat, hoarseness, headache, general malaise, chills, feverishness, and feeling unwell) during 1–4 weeks and 1–8 weeks (Table 6). The changes from baseline level of CD86 and HLA-DR at 4 weeks and CD86 and CD40 at 8 weeks were significantly higher in the LG2055 group (Figure 3 and Supplementary Table 4). No significant differences were observed in pDC frequency and IFN-α production during the intake period (Table 7 and Supplementary Table 5). The results observed in participants aged <40 years were similar to those observed in participants aged <45 years (Supplementary Tables 6–8). Therefore, among young participants, pDC activation and suppression of common cold-like symptoms were observed within 4 weeks of starting ingestion in the LG2055 group.

**Table 6:** Comparison of the cumulative days of each symptom during the intake period (young participants).

Symptoms	Group	1–4 W			1–8 W			1–12 W		
		Without	With	*p*-value	Without	With	*p*-value	Without	With	*p*-value
Runny nose	Placebo	702	194	0.043^*^	1,379	413	0.007^*^	2,115	573	0.053
	LG2055	646	138		1,267	301		1,903	449	
Nasal congestion	Placebo	738	158	0.128	1,476	316	0.180	2,229	459	0.910
	LG2055	668	116		1,319	249		1,954	398	
Sneezing	Placebo	815	81	0.323	1,540	252	1.000	2,270	418	0.667
	LG2055	701	83		1,348	220		1,997	355	
Sore throat	Placebo	734	162	<0.001^*^	1,498	294	<0.001^*^	2,281	407	<0.001^*^
	LG2055	692	92		1,376	192		2,085	267	
Hoarseness	Placebo	796	100	<0.001^*^	1,596	196	<0.001^*^	2,398	290	<0.001^*^
	LG2055	734	50		1,454	114		2,199	153	
Cough	Placebo	813	83	0.131	1,633	159	0.148	2,452	236	0.006^*^
	LG2055	728	56		1,451	117		2,195	157	
Headache	Placebo	804	92	<0.001^*^	1,616	176	<0.001^*^	2,440	248	<0.001^*^
	LG2055	750	34		1,494	74		2,253	99	
General malaise	Placebo	775	121	0.001^*^	1,555	237	0.005^*^	2,361	327	<0.001^*^
	LG2055	717	67		1,410	158		2,135	217	
Chills	Placebo	833	63	0.001^*^	1,670	122	<0.001^*^	2,537	151	<0.001^*^
	LG2055	757	27		1,512	56		2,285	67	
Feverishness	Placebo	854	42	0.008^*^	1,695	97	<0.001^*^	2,559	129	<0.001^*^
	LG2055	766	18		1,532	36		2,308	44	
Fatigue	Placebo	740	156	0.144	1,521	271	0.599	2,320	368	0.137
	LG2055	669	115		1,320	248		1,995	357	
Feeling unwell	Placebo	845	51	<0.001^*^	1,691	101	<0.001^*^	2,550	138	<0.001^*^
	LG2055	769	15		1,531	37		2,297	55	

^*^Significant difference between the two groups (*p* < 0.05).

**Table 7:** Comparison of pDC frequency (stratified analysis).

Week	Group	Young participants (%)		Middle-aged participants (%)	
		Mean ± SD	*p*-value	Mean ± SD	*p*-value
0	Placebo	1.22 ± 0.88	0.588	0.79 ± 0.50	0.505
	LG2055	1.34 ± 0.85		0.85 ± 0.55	
4	Placebo	1.17 ± 0.58	0.838	0.82 ± 0.50	0.846
	LG2055	1.21 ± 0.78		0.84 ± 0.55	
8	Placebo	0.89 ± 0.55	0.162	0.64 ± 0.38	0.524
	LG2055	1.18 ± 0.93		0.68 ± 0.38	
12	Placebo	0.87 ± 0.50	0.871	0.58 ± 0.40	0.180
	LG2055	0.89 ± 0.52		0.68 ± 0.41	

In contrast, the incidence rates of five symptoms (nasal congestion, sneezing, hoarseness, cough, and headache) during the intake period (1–8 and 1–12 weeks) were significantly lower in the LG2055 group than in middle-aged participants. The LG2055 group also showed a significantly lower ratio of “with symptoms” for three symptoms (sneezing, hoarseness, and cough) for 1–4 weeks. The incidence ratios of runny nose and sore throat were significantly higher in the LG2055 group at 1–4 weeks, 1–8 weeks, and 1–12 weeks. No significant differences were observed in the incidence rates of systemic symptoms (Table 8). In addition, changes in HLA-DR from baseline to 8 weeks were significantly lower in the LG2055 group. No other significant differences were observed between the two groups in terms of pDC activity, pDC frequency, and IFN-α production (Tables 7, 9 and Supplementary Table 9).

**Table 8:** Comparison of the cumulative days of each symptom during the intake period (middle-aged participants).

Symptoms	Group	1–4 W			1–8 W			1–12 W		
		Without	With	*p*-value	Without	With	*p*-value	Without	With	*p*-value
Runny nose	Placebo	1,485	223	<0.001^*^	2,898	518	<0.001^*^	4,367	757	<0.001^*^
	LG2055	1,526	350		3,069	683		4,662	966	
Nasal congestion	Placebo	1,539	169	0.080	3,041	375	0.042^*^	4,527	597	<0.001^*^
	LG2055	1,722	154		3,395	357		5,112	516	
Sneezing	Placebo	1,577	131	0.039^*^	3,039	377	<0.001^*^	4,519	605	<0.001^*^
	LG2055	1,765	111		3,449	303		5,131	497	
Sore throat	Placebo	1,589	119	<0.001^*^	3,169	247	<0.001^*^	4,775	349	<0.001^*^
	LG2055	1,654	222		3,398	354		5,095	533	
Hoarseness	Placebo	1,635	73	<0.001^*^	3,261	155	<0.001^*^	4,905	219	<0.001^*^
	LG2055	1,842	34		3,666	86		5,470	158	
Cough	Placebo	1,570	138	<0.001^*^	3,155	261	<0.001^*^	4,722	402	<0.001^*^
	LG2055	1,783	93		3,587	165		5,371	257	
Headache	Placebo	1,619	89	0.703	3,231	185	0.002^*^	4,843	281	<0.001^*^
	LG2055	1,784	92		3,606	146		5,421	207	
General malaise	Placebo	1,646	62	0.269	3,271	145	0.953	4,905	219	0.329
	LG2055	1,794	82		3,594	158		5,409	219	
Chills	Placebo	1,671	37	0.385	3,327	89	0.400	5,009	115	0.643
	LG2055	1,826	50		3,666	86		5,510	118	
Feverishness	Placebo	1,690	18	0.137	3,380	36	0.469	5,069	55	0.999
	LG2055	1,865	11		3,719	33		5,567	61	
Fatigue	Placebo	1,588	120	0.607	3,174	242	0.544	4,745	379	0.151
	LG2055	1,735	141		3,500	252		5,252	376	
Feeling unwell	Placebo	1,666	42	0.751	3,325	91	0.882	5,005	119	0.079
	LG2055	1,826	50		3,655	97		5,466	162	

^*^Significant difference between the two groups (*p* < 0.05).

**Table 9:** Comparison of pDC activity (middle-aged participants).

Marker	Week	Group	Measured value		Change from baseline	
			Mean ± SD	*p*-value	Mean ± SD	*p*-value
CD86	0	Placebo	3,078.0 ± 698.6	0.966	—	—
		LG2055	3,072.9 ± 648.1		—	
	4	Placebo	3,222.3 ± 698.7	0.484	144.3 ± 526.6	0.449
		LG2055	3,143.7 ± 552.9		70.8 ± 567.2	
	8	Placebo	3,416.5 ± 705.5	0.732	338.5 ± 608.9	0.755
		LG2055	3,378.4 ± 529.8		305.5 ± 584.3	
	12	Placebo	3,773.9 ± 770.9	0.277	695.8 ± 643.3	0.240
		LG2055	3,641.6 ± 574.0		568.8 ± 566.5	
HLA-DR	0	Placebo	76,911.6 ± 15,236.7	0.698	—	—
		LG2055	78,043.3 ± 17,673.3		—	
	4	Placebo	80,417.3 ± 16,095.8	0.857	3,505.7 ± 6,418.5	0.628
		LG2055	80,978.6 ± 19,062.1		2,935.4 ± 6,852.4	
	8	Placebo	82,223.5 ± 16,809.6	0.645	5,311.9 ± 6,172.1	0.038^*^
		LG2055	80,833.7 ± 17,170.8		2,790.4 ± 7,392.5	
	12	Placebo	85,556.5 ± 15,591.4	0.958	8,644.9 ± 7,372.6	0.334
		LG2055	85,395.5 ± 18,519.9		7,352.2 ± 7,693.0	
CD80	0	Placebo	3,787.4 ± 666.1	0.307	—	—
		LG2055	3,671.8 ± 603.0		—	
	4	Placebo	3,644.9 ± 517.7	0.531	−142.5 ± 585.5	0.622
		LG2055	3,584.3 ± 574.3		−87.5 ± 672.6	
	8	Placebo	3,692.9 ± 532.0	0.727	−94.4 ± 708.0	0.520
		LG2055	3,658.3 ± 589.9		−13.5 ± 709.0	
	12	Placebo	3,959.8 ± 504.4	0.202	172.5 ± 638.1	0.929
		LG2055	3,833.8 ± 606.1		162.0 ± 687.3	
CD40	0	Placebo	3,729.8 ± 795.5	0.538	—	—
		LG2055	3,804.6 ± 538.5		—	
	4	Placebo	3,834.1 ± 724.2	0.544	104.3 ± 712.0	0.989
		LG2055	3,907.2 ± 624.8		102.6 ± 668.8	
	8	Placebo	4,220.6 ± 820.3	0.989	490.8 ± 780.4	0.577
		LG2055	4,222.5 ± 716.2		417.8 ± 684.7	
	12	Placebo	4,268.7 ± 765.9	0.799	538.9 ± 827.7	0.467
		LG2055	4,235.9 ± 673.0		431.3 ± 840.9	

^*^Significant difference between the two groups (*p* < 0.05).

### 3.5 Safety assessment

In this study, 103 adverse events were reported by 74 participants (45 events in 33 participants in the LG2055 group and 58 events in 41 participants in the placebo group). One adverse event observed in the LG2055 group (body itching) was judged by a medical doctor to be probably associated with the intake of the test samples. A follow-up survey revealed that the severity of the symptom was mild, and the symptom improved rapidly. The relationship between another adverse event observed in the LG2055 group (high creatine kinase value) and the intake of the test samples was inconclusive because we lost contact with the participant, and a follow-up survey could not be performed. The severity of the symptom was mild. All other adverse events were determined not to be, or probably not to be associated with the intake of test samples. The incidence rates during the intake period did not differ significantly between the two groups (33.0% in the LG2055 group vs. 41.0% in the placebo group; chi-squared test, *p* = 0.305). Thus, we concluded that the intake of the test samples did not cause any severe adverse events.

## 4 Discussion

Our previous study revealed that LG2055 activates pDCs (12). LG2055 intake also contributes to the maintenance of physical condition; thus, we hypothesized that the intake of LG2055 suppresses common cold-like symptoms by activating pDCs. However, the detailed relationship between pDC activation and symptom suppression remained unclear. In this study, we conducted an additional clinical trial to investigate pDC activation and maintenance of physical conditions induced by LG2055 intake.

Evaluation of the physical condition using a daily physical health questionnaire showed that the incidence rates of various common cold-like symptoms during the intake period were significantly lower in the LG2055 group (Table 2), consistent with our previous clinical trials (5, 12). The incidence rates of some symptoms did not differ significantly between the two groups. These results suggest that subjective common cold-like symptoms were overall reduced in the LG2055 group.

Subsequently, we investigated the influence of LG2055 intake in young (under 40 years) and middle-aged (40 years and older) participants using a stratified analysis. Compared to the placebo group, the LG2055 group showed significantly higher changes in the expression of cell surface markers such as CD86, HLA-DR, and CD40 from baseline among young participants (Figure 3 and Supplementary Table 4), indicating that LG2055 activated pDCs in young healthy adults. However, significant pDC activation was not observed in the middle-aged participants (Table 9). It has been reported that a decrease in pDC frequency, IFN-*α* production, and the ratio of Toll-like receptor (TLR) 7/9-expressing pDCs with aging were observed (16, 17). Previously, we revealed the mechanisms by which LG2055 induces pDC activation and IFN-α production via TLR9 (10). Considering these reports, it is possible that the relatively low TLR9 expression caused the suppression of pDC activation induced by LG2055 in middle-aged participants. In addition, the pDC frequency during the intake period was higher in the young participants than in the middle-aged participants (Table 7), indicating that the numerical decrease in pDCs might also contribute to age-related suppression of pDC activation. In addition, another cause of pDC dysfunction with aging has been proposed (18), suggesting that aging is an important factor in the immune-stimulating effects of LG2055 intake. In contrast, we previously reported that LG2055 intake suppressed common cold-like symptoms and activated pDCs, particularly in individuals with weakened immune systems (12). Considering our present and previous studies, it is suggested that aging is an important but not the only contributor to distinguishing LG2055 responders; thus, further verification is required to reveal the changes in pDC activation by LG2055 with aging and other factors.

Several strains of LAB, including LG2055, have been reported to activate pDCs and suppress subjective common cold-like symptoms (12–14, 19–21). However, the relationship between pDC activation after LAB ingestion and aging has not been sufficiently elucidated. In this study, we demonstrated that pDC activation induced by LG2055 ingestion was not observed in middle-aged participants and estimated that age-related decreases in TLR9 expression in pDCs may contribute to the suppression of LG2055-induced pDC activation. The detailed mechanisms of pDC activation by LAB have not yet been completely elucidated; thus, TLR9 signaling and other mechanisms may contribute to these effects (10, 22, 23). Additionally, a recent study reported the necessity of phagocytosis in stimulating pDCs by LAB (24); therefore, phagocytosis is considered an important factor in eliciting the immune-stimulating effects of LAB. It is expected that the relationship between pDC activation by LAB and aging will be investigated using multiple strains, and the differences in the results among the strains will be compared, which will contribute to elucidating the detailed beneficial effects of LAB on the host. Furthermore, the relationship between pDC activation by various LAB strains and subjective symptoms should also be elucidated.

Significant differences in pDC activity between the LG2055 and placebo groups in young participants were observed at 4 and 8 weeks after starting intake (Figure 3 and Supplementary Table 4). Although the differences in pDC activity between the two groups became non-significant at 12 weeks, we estimated that the yogurt intake, which served as a placebo, was a contributing factor. It is well known that yogurt intake exerts various beneficial effects on the host, including immune-stimulating effects (25). Focusing on the changes in pDC activity from baseline, pDC activity increased over time during the intake period in both the LG2055 and placebo groups; therefore, it is possible that the placebo induced the activation of pDCs through long-term ingestion. Because significant differences in the changes in pDC activity between the two groups were observed at 4 and 8 weeks, it was estimated that the LG2055-containing active samples induced enhanced pDC activation early in the intake period. In our previous clinical trial, in which the same test samples were used as those in this study, a similar trend was observed for salivary secretory IgA concentrations. A significant difference was observed at 4 weeks but not at 12 weeks (5). Additionally, it is estimated that placebo yogurt intake was effective to a certain extent in reducing physical symptoms; however, yogurt containing LG2055 showed a significantly greater reduction in most physical symptoms compared to the placebo. Therefore, yogurt intake is supposed to improve host health, and the addition of LG2055 to yogurt contributes to early and more beneficial effects.

The present and previous clinical trials have shown that intake of LG2055 for 12 weeks contributes to the suppression of common cold-like symptoms. Furthermore, the evaluation of changes over time revealed that short-term ingestion of LG2055 significantly decreased the incidence of some symptoms. In addition, the number of significantly suppressed symptoms increased with increasing intake duration (Table 2). A similar trend was observed among younger participants (Table 6). Among the young participants, pDC activation was observed at 4 and 8 weeks (Figure 3 and Supplementary Table 4), and the influence of short-term LG2055 intake on subjective physical symptoms was also observed. The decreased incidence rates of symptoms and pDC activation that occurred at similar times suggest a strong possibility that pDC activation induced by LG2055 contributes to the maintenance of the physical condition. However, among middle-aged participants, fewer symptoms showed significant differences in incidence rates compared to young participants; specifically, no significant differences were observed in the incidence rates of systemic symptoms (Table 8). At the same time, the incidence rates of two local symptoms (runny nose and sore throat) were higher in the LG2055 group. The cause of the opposite results in runny nose and sore throat among middle-aged participants was assumed to be attributable to the influence of factors other than infection. For example, mild allergies such as hay fever can induce runny nose and sore throat. In addition, we have shown that the results of the physical health questionnaire can be influenced by chronic symptoms (12). Differences in the backgrounds of the participants might have been partially observed in this stratified analysis. Among the middle-aged participants, several local symptoms were suppressed in the LG2055 group (Table 8); nevertheless, no statistically significant activation of pDCs was observed with LG2055 (Table 9). Activated pDCs stimulate various types of immune cells, inducing an overall immune response and protection from viral infections (11). On the other hand, it has been reported that LABs, including LG2055, can activate some immune cells without involving pDC activation. For example, upregulation of natural killer cell activity (26) and enhanced IgA production by LABs (6) are well known. Therefore, it is estimated that amelioration of common cold-like symptoms could be induced by an enhanced overall immune response via pDC activation and/or upregulation of certain parts of the immune system. In this study, the stratified analysis showed that pDC activation was not observed, and the influence of LG2055 intake on physical condition was weak among the middle-aged participants. It is possible that age-related numerical and functional declines in pDCs induced dysfunction of mechanisms via pDC activation, and that upregulation of parts of the immune system not dependent on pDC activation and limited suppression of physical symptoms were observed. Therefore, it is reasonable to conclude that the influence of LG2055 intake on the physical condition was weak because of the non-significant pDC activation among the middle-aged participants. Indeed, LG2055-induced pDC activation and significant suppression of physical symptoms were observed among young participants with functional pDCs, indicating that pDC activation is important for the effects of LG2055. These results from the stratified analysis support our hypothesis that the intake of LG2055 suppresses common cold-like symptoms by activating pDCs. To enhance the reliability of our hypothesis, further studies and investigations are required.

Although significant pDC activation was observed in the young participants, no significant difference was found between the LG2055 and placebo groups in terms of IFN-α production (Supplementary Table 5). In this study, PBMCs were collected from the participants, stimulated with influenza antigens, and the concentration of IFN-α in the culture medium was measured. This protocol is suitable for substances that enhance pDC sensitivity to antigens. LG2055 directly activates pDCs and induces IFN-α production (10); thus, the protocol applied in this study may not be appropriate for evaluating IFN-α induction by LG2055. Yago et al. (27) reported that lactoferrin, a protein present in mammalian milk, is incorporated by pDCs and enhances their TLR7 response, resulting in the modulation of antiviral immunity against environmental ssRNA viruses. This mechanism of action of lactoferrin would be suitable for evaluation by *ex vivo* stimulation because of its enhanced response to the antigens. It is estimated that direct detection of LG2055-induced IFN-α would be suitable; therefore, further studies are required.

We defined the statistical analysis plan based on other similar studies (5, 12–15, 20, 21); however, multiplicity was not accounted for. Therefore, caution is needed due to the risk of false positives. On the other hand, the results for subjective symptoms showing significant differences between groups were consistent across multiple symptoms (Tables 2, 6). Furthermore, the results obtained in this study were consistent with our previous clinical trials (5, 12). In addition, significant differences in multiple cell surface markers were observed simultaneously in young participants (Figure 3), and pDC activation induced by LG2055 was consistent with previous studies (10, 12); thus, the results and discussion in this study are considered reasonable. When interpreting the results of this study, this limitation should be considered.

Regarding the safety of the test samples used in this study, one adverse event that was probably correlated with the intake of the test samples was observed in the LG2055 group. The reported symptom was “body itching,” and the severity of the symptom was mild. The patient’s symptoms rapidly improved. Food allergies induced by ingredients in the test samples, such as milk, were estimated to cause body itching; however, a detailed investigation was not conducted because of the mild symptoms. The safety of LG2055 intake has been confirmed through various safety tests, including clinical trials (28–30). LG2055 has been used to manufacture commercial dairy products for over two decades, and no safety issues caused by LG2055 have been reported. A mild adverse event that was probably associated with the intake of the test samples was observed in this study; however, there is no need to be cautious about the safety of LG2055. Therefore, continuous safety monitoring of LG2055 is necessary.

Our previous clinical trial, in which the same test samples were used as in this study, was conducted in 2022 (5). This study was affected by the COVID-19 pandemic. Approximately one-fourth of the participants were vaccinated against COVID-19 during the study period, and they implemented infection control measures such as the mandatory use of masks and staying at home. In addition, seasonal influenza epidemics were not observed during the 2021/2022 flu season when the clinical trial was conducted in Japan (31, 32). In the first half of 2025, the lifestyles of the Japanese people were similar to those before the COVID-19 pandemic. None of the participants in the PPS were vaccinated during the study period; thus, we could exclude the influence of vaccination and unusual lifestyle in this study. Moreover, seasonal influenza epidemics were observed in the 2024/2025 flu season in Japan, as well as before the spread of COVID-19. In this study, we confirmed that LG2055 intake suppressed subjective common cold-like symptoms in healthy adults using the same test sample, indicating the usefulness of LG2055 and that its effects were not affected by environmental changes.

## 5 Conclusion

We conducted an additional clinical trial to verify the relationship between the maintenance of physical condition and pDC activation in healthy adults who ingested LG2055. Similar to our previous clinical trials, the intake of LG2055 significantly suppressed the incidence of subjective common cold-like symptoms during the intake period, demonstrating that LG2055 contributes to the maintenance of physical condition in healthy adults. Stratified analysis revealed that LG2055 intake simultaneously activated pDCs and suppressed changes in the physical condition of the young participants. Among middle-aged participants, LG2055 did not activate pDCs, and the influence of LG2055 intake on physical condition was weak. These results indicate that pDC activation induced by LG2055 contributes to the maintenance of physical conditions, supporting the hypothesis that intake of LG2055 reduces subjective symptoms of physical conditions by activating pDCs and improving the host immune system.

## Data Availability

The original contributions presented in the study are included in the article/Supplementary material, further inquiries can be directed to the corresponding author.
