# Supplementary material for: Correlation between plasmacytoid dendritic cell activation and suppression of subjective physical symptoms following Lactobacillus paragasseri SBT2055 ingestion: a randomized, double-blind, placebo-controlled, parallel-group comparative study
Source: Front Nutr. 2026 Jan 12;12:1722081. doi: 10.3389/fnut.2025.1722081 (PMC12832821; doi:10.3389/fnut.2025.1722081)
Supplement: Supplementary file 1 [file Supplementary_file_1.docx]

Supplementary Material

# Supplementary Figures

**Supplementary Figure 1.** The physical health questionnaire. The survey was conducted in Japanese.

# Supplementary Tables

**Supplementary Table 1.** Exclusion criteria.

| No. | Exclusion criteria |
| --- | --- |
| 1 | Participants who regularly consume foods for specified health uses, foods with functional claims, or health foods (including supplements) three or more times per week. |
| 2 | Participants who are unable to discontinue the consumption of foods for specified health uses, foods with functional claims, or health foods (including supplements) from the time consent is obtained. |
| 3 | Participants taking medications (such as antibiotics, intestinal regulators, laxatives, etc.) that may potentially affect the study and who are unable to restrict their use during the study period. |
| 4 | Participants who are currently participating in clinical trials of pharmaceuticals or health foods, have participated within four weeks prior to this trial, or are planning to participate in another trials after the time consent is obtained. |
| 5 | Participants with excessive alcohol intake. |
| 6 | Participants who are unable to discontinue the consumption of foods containing large amounts of lactic acid bacteria or bifidobacteria (such as yogurt, lactic acid bacteria beverages, fermented pickles, including kimchi and nukazuke made with lactic acid bacteria) from the time consent is obtained until the end of the study. |
| 7 | Participants who are aware of having hay fever and regularly take or use medications (excluding eye drops) for hay fever each year, or who are planning to use such medications during the study period. |
| 8 | Night shift workers. |
| 9 | Heavy smokers (21 or more cigarettes per day). |
| 10 | Participants planning to receive vaccinations (for influenza, COVID-19, etc.) from the time consent is obtained until the end of the study. |
| 11 | Participants planning to travel overseas between the time consent is obtained and the end of the study. |
| 12 | Participants with chronic diseases such as diabetes, hypertension, or dyslipidemia who are regularly taking medications. |
| 13 | Participants who are prone to diarrhea after consuming dairy products. |
| 14 | Participants with a previous and/or current medical history of serious diseases of the heart, liver, kidney, digestive organs. |
| 15 | Females who are pregnant or lactating, and those who intend to become pregnant during the trial period. |
| 16 | Participants with allergies to medications and/or food. |
| 17 | Participants who had donated 200 mL of blood or blood components within the last month prior to the initiation of this study. |
| 18 | Males who had donated 400 mL of blood within the last 3 months prior to the initiation of this study. |
| 19 | Females who had donated 400 mL of blood within the last 4 months prior to the initiation of this study. |
| 20 | Males who have had a total of 1200 mL of blood collected within the last 12 months prior to the initiation of this study, including this study. |
| 21 | Females who have had a total of 800 mL of blood collected within the last 12 months prior to the initiation of this study, including in this study. |
| 22 | Participants deemed unsuitable for participation in the trial by the principal investigator or sub-investigator. |

**Supplementary Table 2.** Instructions for the participants.

| No. | Instructions |
| --- | --- |
| 1 | Keep a daily record of the required information during the study period. |
| 2 | Maintain regular lifestyle, including diet and exercise (to avoid overeating, undereating, overexercising, etc.) during the study period. |
| 3 | Avoid the consumption of foods for specified health uses, foods with functional claims, health foods (including supplements), or foods containing large amounts of lactic acid bacteria or bifidobacteria (such as yogurt, lactic acid bacteria beverages, fermented pickles, including kimchi and nukazuke made with lactic acid bacteria) during the study period. |
| 4 | Avoid vaccinations (for influenza, COVID-19, etc.) during the study period. |
| 5 | Inform the designated contacts immediately and keep a record in the event of temporary unwellness or medication use. |
| 6 | Avoid drinking alcohol, overexercising, overeating, and undereating on the day before the test. |
| 7 | Avoid consuming any food and drinks from 21:00 on the day before the test until the end of the test (only water is permitted). |
| 8 | Avoid smoking until the end of the test on the day of the test. |
| 9 | Visit the hospital and undergo examination without having breakfast or consuming the test sample on the day of the test. |
| 10 | Consume the test sample as instructed (one bottle per day). |
| 11 | Discard any surplus test samples properly and do not allow other individuals to consume the test samples. |
| 12 | Keep the information about this study (including the information that provided upon participation) confidential and do not allow other individuals to know. |
| 13 | Avoid donation of blood and/or blood components during the study period. |

**Supplementary Table 3.** Comparison of pDC activity.

| **Marker** | **Week** | **Group** | **Measured value** | | | |  | **Change from baseline** | | | |
| --- | --- | --- | --- | --- | --- | --- | --- | --- | --- | --- | --- |
|  |  |  | **Mean** | **±** | **SD** | ***p* value** |  | **Mean** | **±** | **SD** | ***p* value** |
| CD86 | 0 | Placebo | 3,127.0 | ± | 671.3 | 0.344 |  | - | - | - | - |
|  |  | LG2055 | 3,036.1 | ± | 642.3 |  |  | - | - | - |  |
|  | 4 | Placebo | 3,144.3 | ± | 656.0 | 0.808 |  | 8.7 | ± | 563.0 | 0.334 |
|  |  | LG2055 | 3,122.5 | ± | 563.2 |  |  | 86.3 | ± | 531.4 |  |
|  | 8 | Placebo | 3,374.9 | ± | 657.3 | 0.911 |  | 247.8 | ± | 584.9 | 0.334 |
|  |  | LG2055 | 3,365.1 | ± | 520.4 |  |  | 329.0 | ± | 562.4 |  |
|  | 12 | Placebo | 3,730.4 | ± | 719.5 | 0.368 |  | 603.4 | ± | 611.6 | 0.959 |
|  |  | LG2055 | 3,644.0 | ± | 582.1 |  |  | 607.9 | ± | 594.4 |  |
| HLA-DR | 0 | Placebo | 76,026.4 | ± | 14,846.7 | 0.803 |  | - | - | - | - |
|  |  | LG2055 | 76,602.4 | ± | 16,697.8 |  |  | - | - | - |  |
|  | 4 | Placebo | 78,543.2 | ± | 15,609.7 | 0.544 |  | 2,537.7 | ± | 6,356.3 | 0.337 |
|  |  | LG2055 | 80,050.7 | ± | 18,211.8 |  |  | 3,448.3 | ± | 6,583.0 |  |
|  | 8 | Placebo | 80,741.1 | ± | 16,104.5 | 0.771 |  | 4,714.7 | ± | 6,387.0 | 0.193 |
|  |  | LG2055 | 80,053.4 | ± | 16,242.2 |  |  | 3,450.9 | ± | 6,883.1 |  |
|  | 12 | Placebo | 83,590.0 | ± | 15,283.2 | 0.867 |  | 7,563.5 | ± | 7,510.4 | 0.874 |
|  |  | LG2055 | 83,994.8 | ± | 17,684.5 |  |  | 7,392.3 | ± | 7,245.9 |  |
| CD80 | 0 | Placebo | 3,779.7 | ± | 649.7 | 0.489 |  | - | - | - | - |
|  |  | LG2055 | 3,716.2 | ± | 604.4 |  |  | - | - | - |  |
|  | 4 | Placebo | 3,600.5 | ± | 512.3 | 0.718 |  | −188.5 | ± | 621.3 | 0.635 |
|  |  | LG2055 | 3,572.7 | ± | 542.4 |  |  | −143.5 | ± | 674.4 |  |
|  | 8 | Placebo | 3,655.1 | ± | 563.1 | 0.730 |  | −124.6 | ± | 686.8 | 0.365 |
|  |  | LG2055 | 3,683.2 | ± | 553.2 |  |  | −32.9 | ± | 698.6 |  |
|  | 12 | Placebo | 3,964.3 | ± | 522.3 | 0.433 |  | 184.6 | ± | 616.1 | 0.968 |
|  |  | LG2055 | 3,896.9 | ± | 648.3 |  |  | 180.7 | ± | 714.9 |  |
| CD40 | 0 | Placebo | 3,812.3 | ± | 791.9 | 0.956 |  | - | - | - | - |
|  |  | LG2055 | 3,817.9 | ± | 568.1 |  |  | - | - | - |  |
|  | 4 | Placebo | 3,785.8 | ± | 692.9 | 0.323 |  | −37.2 | ± | 738.5 | 0.322 |
|  |  | LG2055 | 3,881.8 | ± | 629.9 |  |  | 64.0 | ± | 648.7 |  |
|  | 8 | Placebo | 4,198.6 | ± | 790.6 | 0.555 |  | 386.3 | ± | 721.6 | 0.556 |
|  |  | LG2055 | 4,263.8 | ± | 720.4 |  |  | 445.9 | ± | 663.6 |  |
|  | 12 | Placebo | 4,296.5 | ± | 735.3 | 0.700 |  | 484.2 | ± | 736.5 | 0.685 |
|  |  | LG2055 | 4,255.6 | ± | 719.2 |  |  | 437.7 | ± | 833.1 |  |

**Supplementary Table 4.** Comparison of pDC activity (young participants).

| **Marker** | **Week** | **Group** | **Measured value** | | | |  | **Change from baseline** | | | |
| --- | --- | --- | --- | --- | --- | --- | --- | --- | --- | --- | --- |
|  |  |  | **Mean** | **±** | **SD** | ***p* value** |  | **Mean** | **±** | **SD** | ***p* value** |
| CD86 | 0 | Placebo | 3,220.4 | ± | 615.7 | 0.097 |  | - | - | - | - |
|  |  | LG2055 | 2,948.3 | ± | 631.1 |  |  | - | - | - |  |
|  | 4 | Placebo | 2,990.6 | ± | 540.5 | 0.587 |  | −258.1 | ± | 543.9 | 0.004* |
|  |  | LG2055 | 3,071.7 | ± | 594.3 |  |  | 123.4 | ± | 441.0 |  |
|  | 8 | Placebo | 3,295.4 | ± | 555.6 | 0.782 |  | 75.0 | ± | 500.6 | 0.021* |
|  |  | LG2055 | 3,333.5 | ± | 505.3 |  |  | 385.2 | ± | 511.7 |  |
|  | 12 | Placebo | 3,647.4 | ± | 612.5 | 0.988 |  | 427.1 | ± | 510.1 | 0.080 |
|  |  | LG2055 | 3,649.8 | ± | 611.7 |  |  | 701.5 | ± | 657.6 |  |
| HLA-DR | 0 | Placebo | 74,339.1 | ± | 14,154.2 | 0.744 |  | - | - | - | - |
|  |  | LG2055 | 73,154.7 | ± | 13,773.2 |  |  | - | - | - |  |
|  | 4 | Placebo | 74,855.5 | ± | 14,134.1 | 0.456 |  | 633.0 | ± | 5,874.3 | 0.010* |
|  |  | LG2055 | 77,830.3 | ± | 16,100.5 |  |  | 4,675.6 | ± | 5,820.7 |  |
|  | 8 | Placebo | 77,915.4 | ± | 14,499.6 | 0.941 |  | 3,576.3 | ± | 6,730.0 | 0.352 |
|  |  | LG2055 | 78,186.2 | ± | 13,878.5 |  |  | 5,031.6 | ± | 5,261.3 |  |
|  | 12 | Placebo | 79,841.4 | ± | 14,162.6 | 0.835 |  | 5,502.2 | ± | 7,450.3 | 0.264 |
|  |  | LG2055 | 80,643.1 | ± | 15,292.8 |  |  | 7,488.4 | ± | 6,173.0 |  |
| CD80 | 0 | Placebo | 3,765.0 | ± | 627.4 | 0.721 |  | - | - | - | - |
|  |  | LG2055 | 3,822.3 | ± | 605.4 |  |  | - | - | - |  |
|  | 4 | Placebo | 3,513.2 | ± | 498.3 | 0.802 |  | −279.2 | ± | 687.2 | 0.992 |
|  |  | LG2055 | 3,544.8 | ± | 465.7 |  |  | −277.5 | ± | 671.7 |  |
|  | 8 | Placebo | 3,582.9 | ± | 620.7 | 0.257 |  | −182.2 | ± | 651.6 | 0.555 |
|  |  | LG2055 | 3,743.0 | ± | 457.8 |  |  | −79.4 | ± | 683.6 |  |
|  | 12 | Placebo | 3,972.7 | ± | 563.1 | 0.660 |  | 207.7 | ± | 580.9 | 0.922 |
|  |  | LG2055 | 4,047.9 | ± | 729.2 |  |  | 225.5 | ± | 788.4 |  |
| CD40 | 0 | Placebo | 3,969.6 | ± | 773.0 | 0.514 |  | - | - | - | - |
|  |  | LG2055 | 3,849.6 | ± | 642.9 |  |  | - | - | - |  |
|  | 4 | Placebo | 3,690.7 | ± | 627.1 | 0.437 |  | −315.7 | ± | 720.8 | 0.100 |
|  |  | LG2055 | 3,821.1 | ± | 649.6 |  |  | −28.5 | ± | 599.3 |  |
|  | 8 | Placebo | 4,156.6 | ± | 741.5 | 0.285 |  | 187.0 | ± | 551.4 | 0.036* |
|  |  | LG2055 | 4,362.7 | ± | 734.1 |  |  | 513.1 | ± | 616.7 |  |
|  | 12 | Placebo | 4,349.6 | ± | 681.8 | 0.813 |  | 380.0 | ± | 516.5 | 0.689 |
|  |  | LG2055 | 4,302.6 | ± | 831.0 |  |  | 453.0 | ± | 829.2 |  |

* Significant difference between the two groups (*p* < 0.05).

**Supplementary Table 5.** Comparison of IFN-α production (young participants).

| **Week** | **Group** | **Measured value (pg/mL)** | | | |  | **Change from baseline (pg/mL)** | | | |
| --- | --- | --- | --- | --- | --- | --- | --- | --- | --- | --- |
|  |  | **Mean** | **±** | **SD** | ***p* value** |  | **Mean** | **±** | **SD** | ***p* value** |
| 0 | Placebo | 1,264.2 | ± | 1,016.9 | 0.548 |  | - | - | - | - |
|  | LG2055 | 1,125.0 | ± | 763.4 |  |  | - | - | - |  |
| 4 | Placebo | 792.4 | ± | 422.6 | 0.729 |  | −482.3 | ± | 901.1 | 0.377 |
|  | LG2055 | 838.2 | ± | 568.0 |  |  | −286.8 | ± | 784.4 |  |
| 8 | Placebo | 671.0 | ± | 360.6 | 0.487 |  | −593.2 | ± | 1,118.2 | 0.741 |
|  | LG2055 | 608.0 | ± | 337.0 |  |  | −517.0 | ± | 611.4 |  |
| 12 | Placebo | 587.2 | ± | 255.9 | 0.622 |  | −676.9 | ± | 1,024.4 | 0.421 |
|  | LG2055 | 622.0 | ± | 283.1 |  |  | −503.0 | ± | 605.4 |  |

**Supplementary Table 6.** Background characteristics the participants in each group (under 45 years).

|  |  | **Placebo group** | | | **LG2055 group** | | | ***p* value** |
| --- | --- | --- | --- | --- | --- | --- | --- | --- |
|  |  | *n* = 40 | | | *n* = 37 | | |  |
| Sex | male/female | 16/24 | | | 14/23 | | | 1.000 |
| Age | years | 33.5 | ± | 6.2 | 32.5 | ± | 6.4 | 0.506 |
| Height | cm | 163.7 | ± | 7.7 | 164.1 | ± | 7.9 | 0.816 |
| Weight | kg | 56.2 | ± | 9.5 | 57.1 | ± | 10.3 | 0.699 |
| BMI | kg/m^2^ | 20.9 | ± | 2.4 | 21.0 | ± | 2.3 | 0.748 |

Values are presented as mean ± SD.

**Supplementary Table 7.** Comparison of the cumulative days of each symptom during the intake period (under 45 years).

| **Symptoms** | **Group** | **1–4 W** | | | **1–8 W** | | | **1–12 W** | | |
| --- | --- | --- | --- | --- | --- | --- | --- | --- | --- | --- |
|  |  | **Without** | **With** | ***p* value** | **Without** | **With** | ***p* value** | **Without** | **With** | ***p* value** |
| Runny  nose | Placebo | 883 | 237 | 0.283 | 1,720 | 520 | 0.020* | 2,613 | 747 | 0.073 |
|  | LG2055 | 837 | 199 |  | 1,652 | 420 |  | 2,474 | 634 |  |
| Nasal  congestion | Placebo | 929 | 191 | 0.197 | 1,837 | 403 | 0.056 | 2,755 | 605 | 0.022* |
|  | LG2055 | 881 | 155 |  | 1,745 | 327 |  | 2,615 | 493 |  |
| Sneezing | Placebo | 1,006 | 114 | 0.269 | 1,896 | 344 | 0.168 | 2,790 | 570 | 0.006* |
|  | LG2055 | 915 | 121 |  | 1,785 | 287 |  | 2,658 | 450 |  |
| Sore  throat | Placebo | 948 | 172 | 0.029* | 1,923 | 317 | 0.007* | 2,917 | 443 | 0.005* |
|  | LG2055 | 911 | 125 |  | 1,836 | 236 |  | 2,769 | 339 |  |
| Hoarseness | Placebo | 1,017 | 103 | <0.001* | 2,039 | 201 | <0.001* | 3,054 | 306 | <0.001* |
|  | LG2055 | 983 | 53 |  | 1,949 | 123 |  | 2,928 | 180 |  |
| Cough | Placebo | 1,016 | 104 | 0.072 | 2,053 | 187 | 0.098 | 3,078 | 282 | 0.005* |
|  | LG2055 | 962 | 74 |  | 1,927 | 145 |  | 2,905 | 203 |  |
| Headache | Placebo | 999 | 121 | <0.001* | 2,006 | 234 | <0.001* | 3,023 | 337 | <0.001* |
|  | LG2055 | 980 | 56 |  | 1,954 | 118 |  | 2,951 | 157 |  |
| General  malaise | Placebo | 971 | 149 | 0.009* | 1,942 | 298 | 0.006* | 2,941 | 419 | <0.001* |
|  | LG2055 | 936 | 100 |  | 1,853 | 219 |  | 2,810 | 298 |  |
| Chills | Placebo | 1,055 | 65 | 0.015* | 2,114 | 126 | 0.001* | 3,203 | 157 | <0.001* |
|  | LG2055 | 999 | 37 |  | 1,998 | 74 |  | 3,017 | 91 |  |
| Feverishness | Placebo | 1,076 | 44 | 0.003* | 2,141 | 99 | <0.001* | 3,229 | 131 | <0.001* |
|  | LG2055 | 1,018 | 18 |  | 2,035 | 37 |  | 3,057 | 51 |  |
| Fatigue | Placebo | 933 | 187 | 0.288 | 1,902 | 338 | 0.703 | 2,888 | 472 | 0.523 |
|  | LG2055 | 881 | 155 |  | 1,750 | 322 |  | 2,654 | 454 |  |
| Feeling  unwell | Placebo | 1,066 | 54 | <0.001* | 2,129 | 111 | <0.001* | 3,210 | 150 | <0.001* |
|  | LG2055 | 1,018 | 18 |  | 2,020 | 52 |  | 3,030 | 78 |  |

* Significant difference between the two groups (*p* < 0.05).

**Supplementary Table 8.** Comparison of pDC activity (under 45 years).

| **Marker** | **Week** | **Group** | **Measured value** | | | |  | **Change from baseline** | | | |
| --- | --- | --- | --- | --- | --- | --- | --- | --- | --- | --- | --- |
|  |  |  | **Mean** | **±** | **SD** | ***p* value** |  | **Mean** | **±** | **SD** | ***p* value** |
| CD86 | 0 | Placebo | 3,153.4 | ± | 607.3 | 0.118 |  | - | - | - | - |
|  |  | LG2055 | 2,937.0 | ± | 593.1 |  |  | - | - | - |  |
|  | 4 | Placebo | 3,006.1 | ± | 562.5 | 0.342 |  | −168.1 | ± | 539.5 | 0.002* |
|  |  | LG2055 | 3,133.6 | ± | 597.5 |  |  | 196.6 | ± | 430.5 |  |
|  | 8 | Placebo | 3,336.2 | ± | 622.9 | 0.899 |  | 182.8 | ± | 563.6 | 0.054 |
|  |  | LG2055 | 3,352.4 | ± | 488.2 |  |  | 415.4 | ± | 479.7 |  |
|  | 12 | Placebo | 3,674.1 | ± | 770.9 | 0.909 |  | 520.8 | ± | 647.7 | 0.163 |
|  |  | LG2055 | 3,656.4 | ± | 587.9 |  |  | 719.3 | ± | 590.4 |  |
| HLA-DR | 0 | Placebo | 74,532.0 | ± | 13,488.1 | 0.921 |  | - | - | - | - |
|  |  | LG2055 | 74,204.6 | ± | 15,201.6 |  |  | - | - | - |  |
|  | 4 | Placebo | 76,297.2 | ± | 14,238.7 | 0.469 |  | 1,853.0 | ± | 6,352.5 | 0.043* |
|  |  | LG2055 | 78,913.4 | ± | 16,892.3 |  |  | 4,708.9 | ± | 5,729.5 |  |
|  | 8 | Placebo | 78,837.8 | ± | 14,170.6 | 0.956 |  | 4,305.8 | ± | 6,636.1 | 0.744 |
|  |  | LG2055 | 79,027.1 | ± | 15,600.9 |  |  | 4,822.5 | ± | 7,136.0 |  |
|  | 12 | Placebo | 81,122.1 | ± | 13,935.4 | 0.835 |  | 6,590.1 | ± | 7,423.2 | 0.509 |
|  |  | LG2055 | 81,834.3 | ± | 15,724.4 |  |  | 7,629.7 | ± | 6,307.8 |  |
| CD80 | 0 | Placebo | 3,718.9 | ± | 603.3 | 0.503 |  | - | - | - | - |
|  |  | LG2055 | 3,809.5 | ± | 576.8 |  |  | - | - | - |  |
|  | 4 | Placebo | 3,478.8 | ± | 485.8 | 0.317 |  | −260.7 | ± | 648.8 | 0.768 |
|  |  | LG2055 | 3,595.8 | ± | 525.2 |  |  | −213.7 | ± | 730.8 |  |
|  | 8 | Placebo | 3,573.6 | ± | 580.8 | 0.072 |  | −145.3 | ± | 621.6 | 0.360 |
|  |  | LG2055 | 3,804.1 | ± | 529.5 |  |  | −5.4 | ± | 704.8 |  |
|  | 12 | Placebo | 3,932.0 | ± | 555.9 | 0.278 |  | 213.1 | ± | 589.8 | 0.668 |
|  |  | LG2055 | 4,089.5 | ± | 694.2 |  |  | 280.0 | ± | 754.3 |  |
| CD40 | 0 | Placebo | 3,925.3 | ± | 795.4 | 0.566 |  | - | - | - | - |
|  |  | LG2055 | 3,831.1 | ± | 632.0 |  |  | - | - | - |  |
|  | 4 | Placebo | 3,682.7 | ± | 613.6 | 0.459 |  | −270.7 | ± | 707.5 | 0.166 |
|  |  | LG2055 | 3,788.9 | ± | 631.0 |  |  | −42.2 | ± | 716.6 |  |
|  | 8 | Placebo | 4,173.3 | ± | 715.9 | 0.426 |  | 248.0 | ± | 635.0 | 0.159 |
|  |  | LG2055 | 4,302.6 | ± | 702.1 |  |  | 471.5 | ± | 733.6 |  |
|  | 12 | Placebo | 4,379.7 | ± | 680.7 | 0.619 |  | 454.5 | ± | 622.8 | 0.946 |
|  |  | LG2055 | 4,297.2 | ± | 762.1 |  |  | 466.1 | ± | 859.7 |  |

* Significant difference between the two groups (*p* < 0.05).

**Supplementary Table 9.** Comparison of IFN-α production (middle-aged participants).

| **Week** | **Group** | **Measured value (pg/mL)** | | | |  | **Change from baseline (pg/mL)** | | | |
| --- | --- | --- | --- | --- | --- | --- | --- | --- | --- | --- |
|  |  | **Mean** | **±** | **SD** | ***p* value** |  | **Mean** | **±** | **SD** | ***p* value** |
| 0 | Placebo | 1,281.4 | ± | 1,038.7 | 0.786 |  | - | - | - | - |
|  | LG2055 | 1,238.6 | ± | 677.1 |  |  | - | - | - |  |
| 4 | Placebo | 921.5 | ± | 517.1 | 0.968 |  | −359.9 | ± | 976.6 | 0.776 |
|  | LG2055 | 925.8 | ± | 704.1 |  |  | −312.8 | ± | 878.2 |  |
| 8 | Placebo | 668.7 | ± | 377.4 | 0.761 |  | −612.6 | ± | 924.9 | 0.864 |
|  | LG2055 | 649.8 | ± | 316.3 |  |  | −588.8 | ± | 597.0 |  |
| 12 | Placebo | 635.1 | ± | 280.3 | 0.940 |  | −646.2 | ± | 997.9 | 0.796 |
|  | LG2055 | 631.3 | ± | 300.2 |  |  | −607.3 | ± | 639.0 |  |
